# Biogenic silver nanoparticles production and characterization from native stain of *Corynebacterium* species and its antimicrobial activity

**DOI:** 10.1007/s13205-014-0210-4

**Published:** 2014-04-08

**Authors:** B. Gowramma, U. Keerthi, Mokula Rafi, D. Muralidhara Rao

**Affiliations:** Department of Biotechnology, Sri Krishnadevaraya University, Anantapuram, 515003 Andhra Pradesh India

**Keywords:** Diamine silver, Silver nanoparticles, Native strain, EDX, TEM, SEM, Antimicrobial activity

## Abstract

In the present study, synthesis, characterization, and the antibacterial activity of silver nanoparticles from native isolate of *Corynebacterium glutamicum* has been reported. Silver nanoparticles were synthesized by challenging the dried biomass of *C. glutamicum* with aqueous diamine silver ([Ag (NH_3_)^2^]^+^) containing 1 mM AgNO_3_. Synthesized silver nanoparticles (AgNPs) were characterized by ultraviolet–visible spectroscopy and energy-dispersive X-ray (EDX) spectroscopy analysis. Morphological study of silver nanoparticles was carried out using transmission electron microscopy (TEM) and scanning electron microscope (SEM). The spherical morphology of silver nanoparticles was confirmed from SEM image. The TEM image showed the average particle size of silver nanoparticles was about 15 nm. Silver nanoparticles synthesized from *C. glutamicum* were found to have enhanced antimicrobial activity against selected pathogenic strains. Silver nanoparticles from pure strains of *Corynebacterium* species was done by many investigators, but as per the present literature, this is the first report on the production of silver nanoparticles using a native strain of *Corynebacterium*.

## Introduction

Nanotechnology is referring to the ability for designing, characterization, production and application of structures, devices and systems by controlling shape and size at the nano scale (Mansoori et al. 2007) and it is a promising emerging industry, which is bringing us exiting new products. Currently, there is a growing need to use environmental friendly nanoparticles that do not produce toxic wastes in their synthesis protocol (Vahabi et al. 2011). Many methods have been designed to synthesize nanoparticles and the most important aspects of nanotechnology rely on the synthesis of nanoparticles with well-defined sizes, shapes and controlled monodispersity (Pugazhenthiran et al. 2009). Biotechnological route has emerged as a safe and alternative process in the synthesis of nanoparticles by employing ambient biological resources (Baker et al. 2013). Studies have reported that the biological methods depict an inexpensive and eco-friendly route for synthesis of nanoparticles. Synthesis of nanoparticles have been demonstrated from some microbes, among them the bacterial species used have included *Escherichia coli* (Natarajan et al. 2010), *Bacillus cereus* (Sunkar and Nachiyar 2012), *Corynebacterium* sp. (Arun et al. 2013), while yeast species have included MKY3 yeast strain (Kowshik and Ashataputre, 2003), *Saccharomyces cerevisiae* BU-MBT CY-1 (Selvakumar et al. 2011), fungi included *T. asperellum* (Mukherjee et al. 2008), *Aspergillus clavatus* (Verma et al. 2010) *Trichoderma Reesei* (Vahabi et al. 2011), *Aspergillus terreus* (Li et al. 2012), algae *Cyanobacteria* (Sudha et al. 2013) and lichen *Parmotrema praesorediosum* (Mie et al. 2013) are able to absorb and accumulate metal and can be used in the reduction of environmental pollution and also for the recovery of metals from waste. The adaptation to heavy metal-rich environments is resulting in microorganisms that express activities, such as biosorption, bioprecipitation, extracellular sequestration, transport mechanisms, and chelation and such resistance mechanism forms the basis for the use of microorganisms in production of nanoparticles (Baker et al. 2013).

Among the noble metals, silver (Ag) is the metal of choice in the field of biological system, living organisms and medicine (Parashar et al. 2009). Many studies have proved that microorganisms can produce nanoparticles either by enzymatic or non-enzymatic reduction mechanism. Ahmad et al. (2003) had shown that NADH-dependent enzymes are responsible for the biosynthesis of nanoparticles. Few researchers reported that nanoparticles were produced without the involvement of biological enzymes. Liu et al. (2000) produced Au3+ nanoparticles from dried cells of *Bacillus megaterium.* Studies on the absorption of Ag+ by some microorganisms have been reported (Sneha et al. 2010). In these cases, no involvement of enzymes was observed; this non-enzymatic reduction mechanism suggested that some organic functional groups of microbial cell walls could be responsible for the production process under certain conditions (Lin et al. 2001). Dried biomass of some microorganisms, such as *Lactobacillus* A09, *Bacillus megaterium* D01 also has the ability to reduce Ag+ ions through the interaction between Ag+ and some groups on the microbial cell walls (Fu et al. 2000).

When compared with all the other types of nanomaterials silver nanoparticles have proved to be the most effective antimicrobial agents also they have shown great promise in terms of biomedical applications, not only due to their large surface area to volume ratio (Bhattacharya and Mukherjee 2008; Hirst et al. 2009), but also different biomedical activities (Hussain and Ferguson 2006). In particular, because of the recent advances in research on metal nanoparticles, Ag-NPs have received special attention as a possible antimicrobial agent (Baker et al. 2005; Firdhouse et al. 2013). A recent study showed that yeast and *E. coli* was inhibited at a low concentration of AgNPs, the study of mechanisms revealed that free radicals and oxidative stress was responsible for the antibacterial activities (Kim et al. 2007). Disease causing microbes that have become resistant to drug therapy are an increasing public health problem. Therefore, there is a vital need to develop new bactericides.

Current work was focused on the synthesis and characterization of silver nanoparticles from native isolate of *Corenebacterium glutamicum* by non-enzymatic method and the assessment of antibacterial activity against pathogenic bacteria.

## Materials and methods

### Chemicals

Peptone, beef extract, yeast extract, bacto tryptone, agar agar, potato dextrose, silver nitrate (AgNO_3_), NH_3_·H_2_O (25 % w/w, AR), NaOH, NaCl, HNO_3_, etc.

### Bacterial culture for silver nanoparticles production

The bacterial strain *C. glutamicum* was isolated from native soil and characterized performing biochemical tests. The strain was maintained at 4 °C on nutrient agar slants as well as sub cultured from time to time to regulate its viability. *C. glutamicum* is a small, non-motile, gram-positive soil bacterium. It is non-pathogenic, non-spore forming, grows quickly, has relatively few growth requirements, has no extracellular protease secretion, and used to produce many amino acids.

### Microbial cultures to test antimicrobial sensitivity

Bacterial strains *Staphylococcus aureus* MTCC3160, *E. coli* MTCC40*, Salmonella enterica* MTCC3917, *Pseudomonas aeruginosa* MTCC424, *Klebsiella pneumoniae* MTCC3384, and *Shigella flexneri* MTCC1457 were procured from Institute of Microbial Type Culture Collection (MTCC), Chandigarh, India and *Bacillus subtilis, Bacillus flexus* were isolated from the native soil of Sri Krishnadevaraya University, Anantapuram, AP India and the cultures were maintained at 4 °C on nutrient agar slants.

### Preparation of diamine silver

Diamine silver complex ([Ag(NH_3_)^2^]^+^) was prepared by adding dilute ammonia solution (NH_3_·H_2_O, 25 % w/w, AR) into aqueous solution of silver oxide (Ag_2_O) until the precipitate of Ag_2_O was transformed into soluble [Ag(NH_3_)_2_]^+^ (Ag when treated with alkali AgNO_3_ forms silver oxide, which in case of NH_4_OH dissolves to form complex ion) (Vogel 1956).

### Production of biomass

*C. glutamicum* cultures were maintained by subculturing at monthly intervals and growth conditions were optimized. Luria Broth (LB) (1 % bactotryptone, 0.5 % yeast extract, 1 % NaCl, pH 7.0 ± 0.2) was used for growing the organism. 250 ml of LB was prepared using Milli-Q water, autoclaved at 121 ± 1 °C for 15 min and inoculated with a fresh batch of the bacteria, *C. glutamicum.* The culture flasks were incubated for 72 h at 30 °C with shaking at 120 rpm. After 72 h of growth, the biomass were harvested by centrifugation at 5,000 rpm for 10 min and the collected cell pellet was washed three times with deionized water to remove the culture medium and dried overnight in an oven at 60 °C. Silver nitrate will form precipitation with the salts present in the water, to avoid that Milli-Q water is used throughout the experiment.

### Synthesis of Ag nanoparticles from *C. glutamicum* biomass

*C. glutamicum* at the concentration of 0.5 g/l dried biomass was resuspended with deionized water and added to aqueous solutions of freshly prepared 15 ml of a diamond silver complex containing 1 mM silver nitrate and made final volume to 25 ml with deionized water then incubated for 24 h at room temperature in a orbital shaker at 150 rpm. Control (without the diamine silver, only biomass) was also run along with the experimental conditions. After 24 h of incubation, 1 ml sample was with drown and centrifuged. Then, the supernatant was assayed for of silver nanoparticle formation. The amount of silver nanoparticles produced by *C. glutamicum* was determined by adopting the method recommended by Zhang et al. (2005). The concentration of Ag in the supernatant was (Q, mg g^−1^) calculated as:

Q=[(Ci-Cb)V]/M

where V was the volume of sample solution (L), Ci and Cr were the initial and equilibrium Ag concentration (mg/l) in solution, respectively, and M was the weight of dried biomass (g).

### UV–Vis spectrophotometer analysis

UV–Vis analysis of the reaction solutions was carried out at room temperature using Thermo scientific-EVOLUTION201 spectrophotometer equipped with matching quartz cells at a resolution of 1 nm from 200 to 800 nm. The supernatant was diluted with deionized water (1:1) and the absorption was measured. It is generally recognized that UV–Vis spectroscopy could be used to examine the size and shape controlled nanoparticles in aqueous suspensions (Wiley et al. 2006).

### Transmission electron microscopy (TEM)

TEM grids were prepared by sonicating the AgNPs sample solution for 5 min and placing a few drops on the 300 mesh carbon-coated copper grid and dried for the complete evaporation of water under a lamp and operated at an accelerating voltage of 200 kV using EM2000Fx-II, transmission electron microscope, which is a 200 kV HRTEM from JEOL, Japan, to characterize the sample after usual alignment procedures. In situ LCD camera is used to record the pictures.

### Scanning electron microscope (SEM)

Sample for scanning electron microscopic (SEM) analysis was prepared as thin films on a carbon-coated copper grid by just dropping a very small amount of the sample on the grid, extra solution was removed using a blotting paper and then the film on the SEM grid were allowed to dry by putting it under a mercury lamp for 5 min. The instrument used is a JEOL 840 with resolution at 20 kV: 10 nm.

### Energy-dispersive X-ray (EDX) analysis

Energy-dispersive X-ray spectrometers take advantage of the photon nature of light. EDX analysis was performed by measuring the energy and intensity distribution of X-ray signals generated by a focused electron beam on a specimen operating at 120 kV using ESEM Quanta 200, FEI EDX instrument. The data are used to obtain the elemental composition of the material.

### Antimicrobial assay

Pure cultures of bacteria to be tested were grown in nutrient broth for 24 h at room temperature. In this method, sterile nutrient agar plate was prepared. Bacterial pathogens used in the present experiment were spread over the agar plate using sterile spreader. The plates were allowed to dry and using a sterile well-cutter with the diameter of 6.0 mm two wells were made in each agar plate, one for control (labeled as C) and the other for test (labeled as C). Subsequently, 30 μl (5 μg) of nanoparticles suspension was introduced into the wells labeled as ‘T’ another well left empty without adding silver nanoparticles maintained as a control of the inoculated nutrient agar plates and then incubated at 25 °C for 24 h, measured the diameter of inhibitory zones in mm after incubation (Lee et al. 2010). The assays were performed in triplicates and the mean values were recorded.

## Results and discussion

**Fig. 1:**
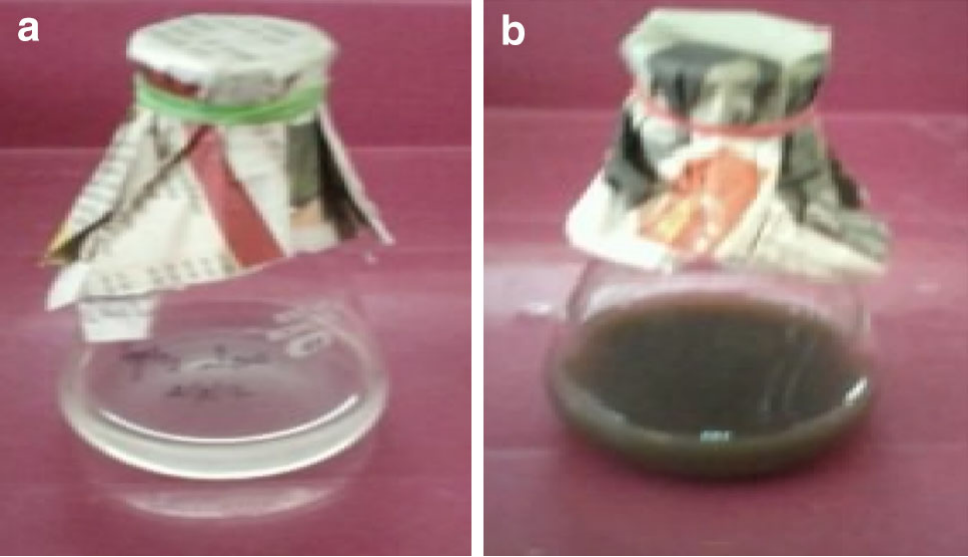
*C. glutamicum* biomass containing diamine silver before (**a**) and after (**b**) production

Silver nanoparticles were synthesized successfully from dried cells of native *C. glutamicum* with aqueous diamine silver solution. The presence of nanoparticles in the medium was confirmed by the change in color from colorless to brown or deep yellow shown in Fig. 1b, where control (without diamine silver) showed no color formation in the culture when incubated for the same period and conditions (Fig. 1a). The color intensity increased with a period of incubation due to the reduction in silver nanoparticles. The characteristic brown color of colloidal silver solution is due to the excitation of surface plasmon vibrations in the nanoparticle and provides a convenient spectroscopic signature of their formation (Saifuddin et al. 2009). The yield of silver nanoparticles from native strain of *C. glutamicum* was found good and the concentration of silver nanoparticles present in the supernatant was estimated 280 mg/gram dried biomass.

### UV–Vis spectrophotometer analysis

**Fig. 2:**
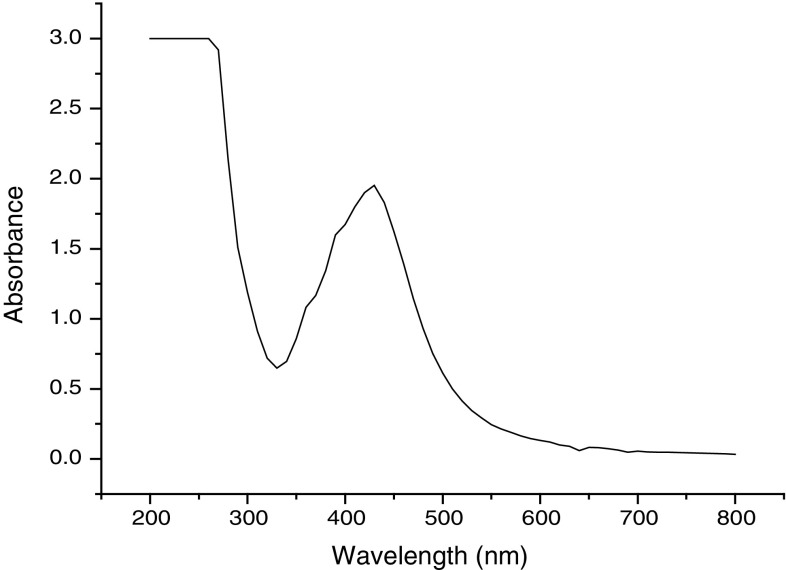
UV–Vis spectra of silver nanoparticles showing maximum absorbance at 450 nm

The silver nanoparticles were characterized by UV–Vis spectroscopy, one of the most widely used techniques for structural characterization of silver nanoparticles (Sun et al. 2001). Biological method of silver nanoparticles synthesis exhibit strong absorption of electromagnetic waves in the visible range due to their optical resonant property, called occurs due to its collective oscillation of conduction electrons, combined with the incident light (Kreibig and Vollmer 1995). Figure 2 shows the UV–Vis absorption spectra of the 96 h old AgNPs sample at 440 nm. The spectra recorded from the *C. glutamicum* reaction vessel at different reaction times and observed increased intensity in absorption spectra of silver solution with time, indicating the formation of increased number of silver nanoparticles in the solution (the graph is not included); this is similar to the findings of Esumi et al. (2000), Zhang et al. (2005). The prepared aqueous solution of AgNPs showed a strong absorption band between 410 and 440 nm, which is a typical absorption band of spherical Ag nanoparticles due to their surface plasmon.

### TEM analysis of silver nanoparticles

**Fig. 3:**
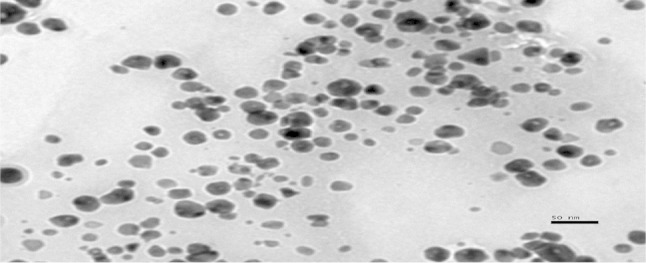
TEM image of synthesized AgNPs

The well-known technique for imaging solid materials at atomic resolution is TEM. The technique was employed to visualize the size and shape of Ag nanoparticles. From the results, it is observed that most of the Ag nanoparticles were spherical in shape. Figure 3 shows the TEM image of individual silver nanoparticles with the mean particle size estimated as 15 nm.

### SEM analysis

**Fig. 4:**
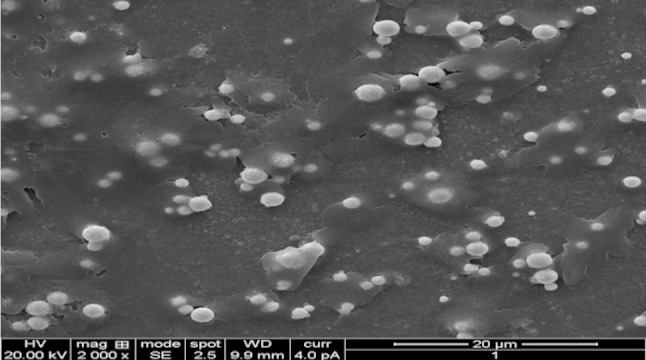
SEM image of silver nanoparticles

The scanning electron microscopy has been employed to characterize the size, shape and morphologies of forming silver nanoparticles. The SEM image of the sample was shown in Fig. 4. From the results morphology of AgNPs was more clearly seen, the particle shapes were circular and poly-dispersed and the size ranged between 15 and 20 nm. The size of obtaining nanoparticles was less when compared with the nanoparticle size of Arun et al. (2013), where they got diameter ranged from 24.55 to 32.02 nm, but similar with the results (Zhang et al. 2005). A report by Gurunathan et al. (2009a) showed that by controlling the environment of nanoparticle synthesis, silver nanoparticles of various sizes and shapes could be synthesized. At acidic pH, the size of the nanoparticles ranged 45 nm, whereas at pH 10 the size is just 15 nm. It is also due to increase in size of the particles with growth time (Saha et al. 2011).

### EDX studies

**Fig. 5:**
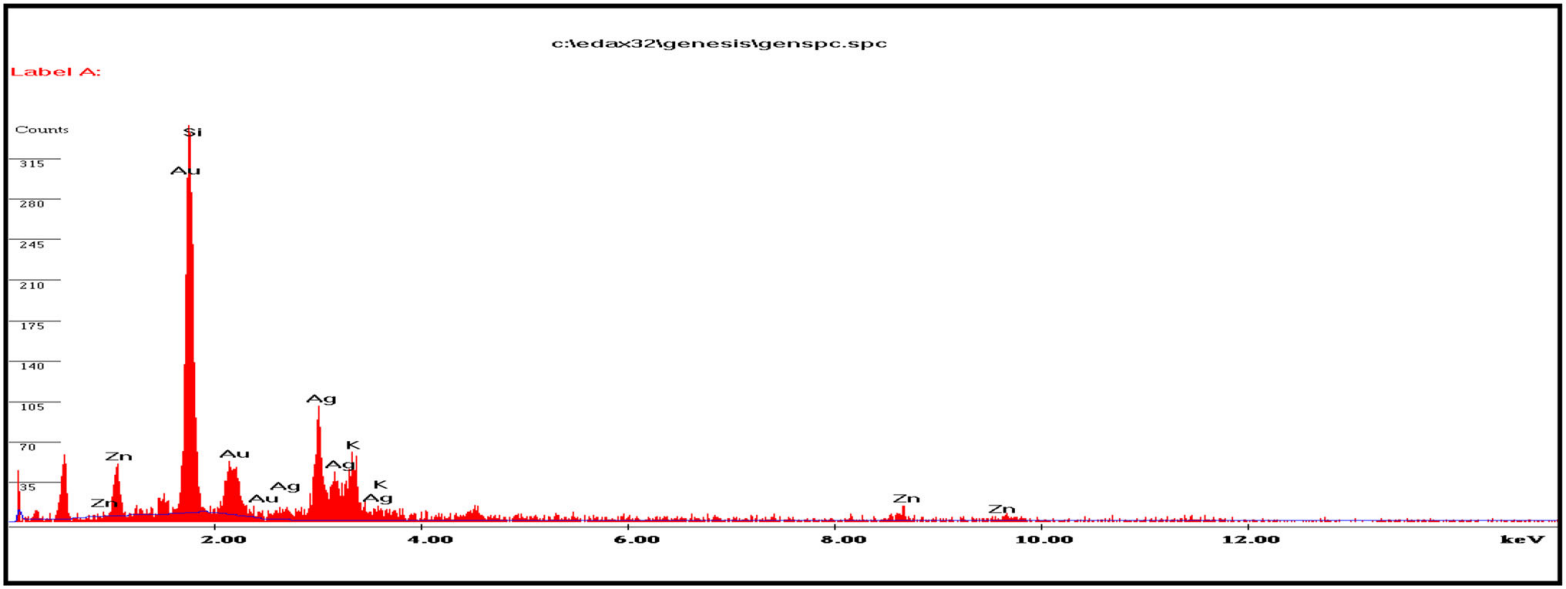
Energy-dispersive X-ray spectrum of nanoparticles

EDX spectrum of silver nanoparticles was checked and was found to contain a great deal of silver. Figure 5 shows strong peaks from the silver atoms in the nanoparticles are observed at 2.6, 3.0, 3.2, and 3.6 keV. The presence of the optical absorbance band at ~3 eV reveals the presence of pure metallic silver nanoparticles (Magudapathy et al. 2001). The graph also shows the presence of zinc (Zn), potassium (K)and aurum (Au) in the EDX picture of silver nanoparticles. This is probably due to the presence of substrate over which the NP sample was held during SEM microscopy.

### Antimicrobial activity

**Fig. 6:**
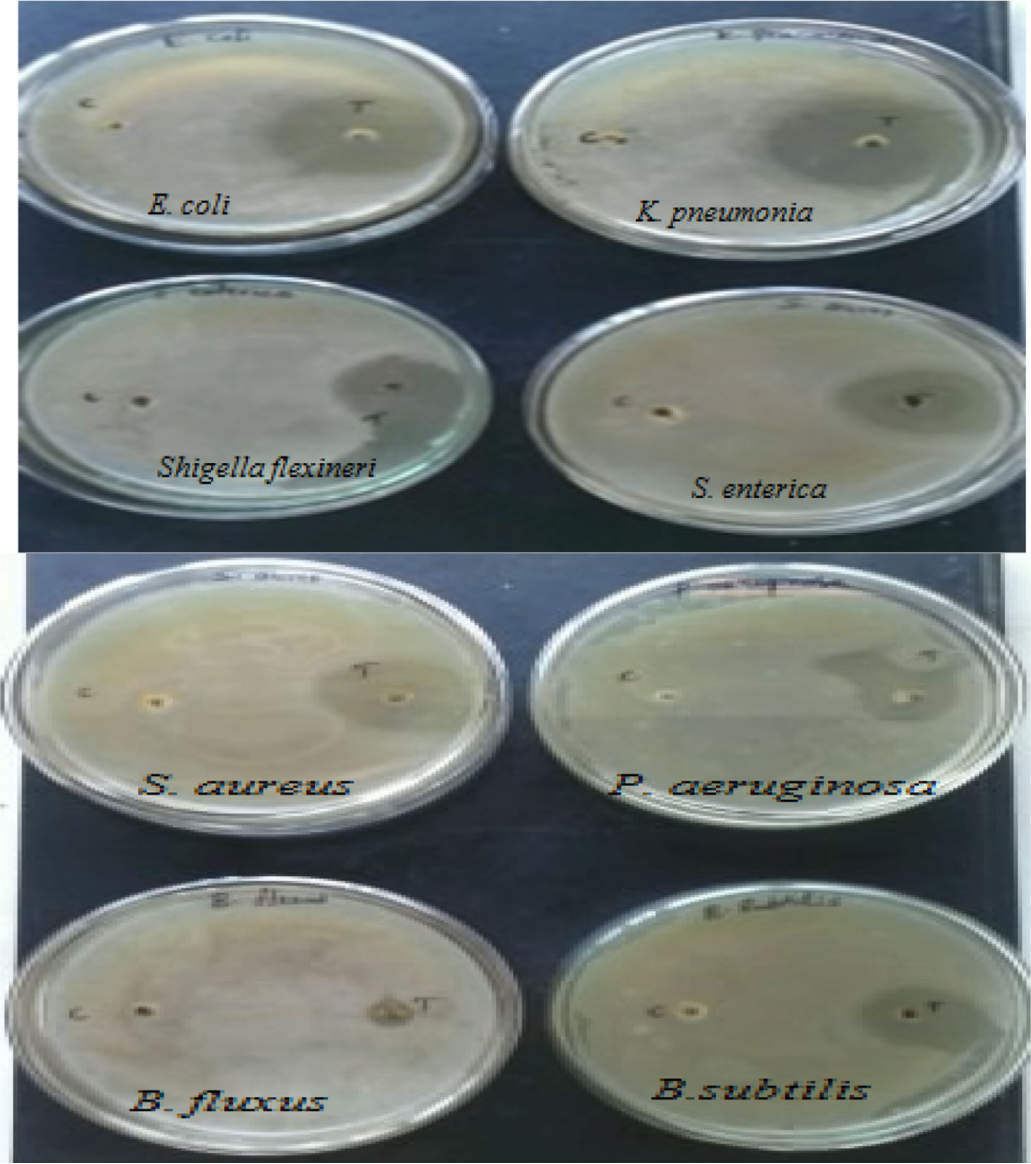
Antimicrobial activity of silver nanoparticles against bacterial pathogens

Microbial contamination of air, water and soil due to different types of microorganisms creates problems in living conditions, in the public health and industrial fields. As a result, an increased occurrence of antibiotic resistant genes in many bacterial species is found in human beings and animals (Raffi et al. 2010). Emergence of new bacterial strains resistant to current antibiotics has become a serious public health issue, which raised the need to develop new bactericidal materials. Utilization of silver as a disinfecting agent is not new, and silver compounds were shown to be effective against both aerobic and anaerobic bacteria by precipitating bacterial cellular proteins and by blocking the microbial respiratory chain system (Gravante et al. 2009; Monteiro et al. 2009). In the present study, 5 μg of the nanoparticles was taken as final products for antimicrobial assay. The antimicrobial activity of the synthesized silver nanoparticles was studied against *E. coli, Klebsiella pneumonia*, *Shigella flexneri, Salmonella enterica, Staphylococcus aureus, Pseudomonas aueroginosa, and Bacillus flexus Bacillus subtilis* using well diffusion technique (Fig. 6) and the assay was performed in triplicates. Test and control were maintained in all the plates. In the control zone of inhibition was not observed. In the test zone, inhibitory zone was clearly observed and the mean diameter of inhibitory zones around each well with AgNPs is measured. From the mean values, highest antimicrobial activity was observed against *K. pneumonia*, which was 18 mm in diameter. Although the cultures of *E. coli, S. aureus, S. enterica, S. flexneri, P. aueroginosa, B. subtilis* and *B. flexus* were also showed clear zones of inhibition, which were about 16, 15, 15, 14, 12, 8 and 4 mm in diameter, respectively. Many other studies (Durán et al. 2007; Shirley et al. 2010; Tollamadugu et al. 2011; Arun et al. 2013; Firdhouse and Lalitha 2013; Madhavaraj et al. 2013) proved the antimicrobial activity of AgNPs and the nanoparticles from native strain have shown comparatively good inhibitory zone.

## Conclusion

Silver nanoparticles from novel *C. glutamicum* strain were successfully synthesized, and the characterization of the nanoparticles was carried out using UV–Vis spectroscopy, TEM, SEM, and energy-dispersive X-ray analysis. Native *C. glutamicum* strain is greatly reduced diamine silver into silver nanoparticles at the concentration of 280 mg/g dried biomass. Synthesized nanoparticles were showing surface plasmon resonance (SPR) between 410 and 450 nm. The TEM analysis indicates the presence of 15 nm size of silver nanoparticles. SEM images clearly evident that the synthesized silver nanoparticles were spherical in shape. Chemical analysis performed with the use of EDX revealed that the powder contains of silver. The antimicrobial potential of synthesized Ag nanoparticles was tested against infectious bacteria by well diffusion assay. The AgNPs was showing strong antimicrobial potential against the tested bacteria. The present study is eco-friendly need cheaper cultivation requirements, higher growth rates on laboratory scales. Present findings not only confirm the nanoparticle formation, but also implicate the efficient antimicrobial property of silver nanoparticles.
